# Solitary Plasmacytomas: Current Status in 2025

**DOI:** 10.3390/hematolrep17040032

**Published:** 2025-06-30

**Authors:** Uğur Hatipoğlu, Mert Seyhan, Turgay Ulas, Mehmet Sinan Dal, Fevzi Altuntaş

**Affiliations:** Department of Hematology & Apheresis Unit, Ankara Oncology Training and Research Hospital, University of Health Sciences, Ankara 06170, Türkiye; ugurugur2192@gmail.com (U.H.); drseyhanmert@gmail.com (M.S.); dr.sinandal@gmail.com (M.S.D.); faltuntas@hotmail.com (F.A.)

**Keywords:** plasmacytoma, plasma cell tumor, multiple myeloma, bone, extramedullary

## Abstract

Solitary plasmacytoma refers to a neoplastic, clonal proliferation of plasma cells forming a single mass. They are divided based on their origin site; solitary bone plasmacytomas originate from the bones, and extramedullary plasmacytomas represent extraosseous tumors. These are rare tumors but carry a risk of transforming to multiple myeloma; thus, optimal management and meticulous follow-up are needed. Their rarity poses a major challenge in conducting large-scale clinical trials, leaving important gaps in evidence regarding best practices. Newer imaging techniques have improved the quality of staging, management decisions, and outcomes. Radiation still has a significant role in treatment algorithms, and adjuvant chemotherapy is gaining more importance; trials are underway in this area. Follow-up should contain biochemical tests as the proposed response definition criteria. We aimed to review the key studies and guidelines in this paper.

## 1. Introduction and Epidemiology

Solitary plasmacytomas (SPs) are plasma cell tumors characterized by the localized proliferation of malignant cells and carry the risk of progression to overt myeloma within 17 to 120 months, according to various studies. They are quite uncommon, representing 5% of all plasma cell neoplasms. Solitary bone plasmacytomas (SBPs) are more common than extramedullary plasmacytomas (EMPs) at a ratio of approximately 2 to 5:1, and males are nearly twice as affected as females. The median age at diagnosis ranges from 55 to 60, with almost one-third of patients being under the age of 50. Plasmacytomas can affect many parts of the body, presenting various symptoms and findings. The clonal plasma cell percentage in bone marrow should be lower than 10%, and there should be no additional lesion on cross-sectional imaging and end-organ involvement (anemia, hypercalcemia, kidney injury, etc.) by diagnostic definition (Table 1 and Table 2). Up to 10% of clonal plasma cells in the marrow are regarded as solitary plasmacytoma with minimal bone involvement. The absence of end-organ manifestations and no more than 10% plasma cells in marrow can easily differentiate plasmacytomas from smoldering or active multiple myeloma. Macrofocal-type myeloma is an important differential diagnosis, easily distinguished from solitary plasmacytoma by the presence of multiple lytic bone lesions [1,2,3,4,5]. Imaging studies, ranging from radiographs to FDG-PET CT, assist in diagnosis and also have some prognostic implications [6,7,8]. Management and follow-up principles differ from those for multiple myeloma and other plasma cell neoplasms; while a minority of patients can be cured by surgery alone, some require a more aggressive approach. Drug therapy for pure solitary plasmacytoma is a significant topic of debate, and the optimal radiation dose must also be addressed [9,10,11,12,13].

In this manuscript, we review the current literature and synthesize the landmark studies and clinical trials alongside the most recent reports. We aim to provide a state-of-the-art overview of solitary plasmacytomas for practicing hematologists and oncologists, addressing the notable lack of comprehensive and up-to-date works on this challenging condition.

## 2. Diagnostic Evaluation

### 2.1. Clinical Features

SPs have variable manifestations dictated by the origin site. SBPs can cause pathological fractures, radiculopathy due to nerve root compression, and tumor formation by itself or amyloid accumulation [1]. The axial skeleton is more commonly affected than the appendicular skeleton, and the vertebrae are the most common site for the SBP [13]. EMPs have been reported for almost every tissue, and the clinicoanatomic spectrum for them is enormous. Underlying EMP could present with a superior vena cava clot when stemming from mediastinal structures, a breast lump, or gastric outlet obstruction [2,3]. As a result, EMP diagnosis is often diagnostically challenging, needs detailed evaluation, and requires multidisciplinary expert involvement. Anemia, hypercalcemia, and renal impairment are typical for overt myeloma, not plasmacytoma [1].

### 2.2. Imaging

Imaging studies are the cornerstone of diagnosis and follow-up. Magnetic resonance imaging (MRI) and fluorodeoxyglucose positron emission tomography (FDG-PET CT) are the most common techniques to detect plasmacytomas. Plain radiographs and computed tomography generally serve as initial studies to address patients’ complaints.

#### 2.2.1. Plain Radiographs and Computed Tomography

Plain radiography is easy to obtain and has been used for a very long time [6]. In addition to the initial evaluation of a patient with musculoskeletal complaints, plain radiographs have been used as a skeletal survey, obtained after myeloma diagnosis, to assess lytic bone lesions or concurrent bone plasmacytomas. SBP has a lytic appearance on radiographs, called a soap-bubble appearance [14,15]. There is no surrounding sclerosis, and a thin zone separates the tumor from healthy bone; SBPs frequently replace trabecular bone [16,17]. However, skeletal surveys lack sensitivity. At least 30% of trabecular bone loss is required to detect osteolytic lesions, which could lead to misdiagnosis of SP instead of active multiple myeloma [1]. Sclerotic lesions are not a usual finding, and their presence may be a sign of POEMS syndrome [18]. Giant cell tumor and aneurysmal bone cysts should be considered when cyst formation is present [19]. Plain X-ray is not sensitive for EMPs [20].

Computed tomography (CT) plays a crucial role in the anatomical localization and morphological assessment of extramedullary plasmacytomas. On CT, EMPs are characterized by well-defined and hypo/isodense lesions relative to surrounding tissues during the arterial phase [3]. However, these are non-specific and can mimic a wide array of neoplastic and non-neoplastic entities [3]. In the context of multiple myeloma, the presumptive diagnosis of EMP is straightforward, but the absence of such a diagnosis always warrants histopathologic confirmation [3]. Due to its widespread availability, a CT scan would be useful for detecting spinal cord compression if an MRI scan is not readily available [20]. For SBPs, the extent of lesion and cortical outline delineation can be accurately assessed with CT [15]. However, MRI and PET CT are superior for detecting focal bone lesions and are preferred over CT [15].

#### 2.2.2. MRI

On MRI, SBPs appear as disruptions in the marrow signal caused by marrow replacement. On T1-weighted images, the signal intensity is similar to that of muscle. They show enhancement with gadolinium on T2W images [1]. Between 25% and 60% of SBPs are found in the spine. Some degree of vertebral collapse may be seen when the spine is involved. The mini-brain appearance, characterized by T1W and T2W hypointense struts, is highly specific for bone plasmacytoma, whereas hemangiomas are the main differential [15]. Whole-body MRI (WB-MRI) has become the new gold standard imaging modality for plasma cell neoplasms, including multiple myeloma [21]. The core Myeloma Response Assessment and Diagnosis System (MY-RADS) protocol has been published [22], and this system includes (a) sagittal whole spine T1W and fat-suppressed T2W sequences, (b) axial whole-body diffusion-weighted imaging, and (c) axial whole-body T1W Dixon sequences. Sagittal whole spine T1W and T2W sequences are used to evaluate spinal anatomy, compression fractures, and neural compromise, while diffusion-weighted studies can provide information about the functional status of focal or diffuse lesions [22]. MRI is a sensitive tool for assessing the risk of progression to myeloma, as Liebross et al. demonstrated that a significantly higher number of radiograph-sine-evaluated patients progressed to overt myeloma [14]. The International Myeloma Working Group also mandates the exclusion of additional lesions for the diagnosis of SP [23]. WB-MRI is recommended by the 2025 guidelines of the National Comprehensive Cancer Network (NCCN) for the evaluation of SBPs [24].

#### 2.2.3. PET CT

Differential diagnosis of myeloma and solitary plasmacytoma is crucial, because the management strategies differ significantly between these entities. Florine-18-fluorodeoxyglucose PET CT (18-FDG-PET CT) is an important imaging technique assessing plasma cell neoplasms, but its role in solitary plasmacytomas has not been completely established [20]. FDG-PET CT serves as a diagnostic tool and a prognostic indicator as well [20]. Most SPs are FDG-avid lesions, and FDG-avidity is best correlated with tumor diameter [25,26]. In their review, Albano et al. clearly showed the diagnostic power and sensitivity of FDG-PET [25]. In a prospective trial, 18-FDG-PET CT performed better than bone scintigraphy and CT in terms of detecting additional lesions [27]. Nanni et al. retrospectively investigated 14 patients to compare the diagnostic accuracy of 18-FDG-PET CT with conventional imaging (MRI and plain radiography). The 18-FDG-PET CT study was more successful than MRI: 6 of 14 patients were upstaged to myeloma, and all cases were SBPs [26]. Different radiotracers other than fluorodeoxyglucose, such as fluorocholine, have been tested in myeloma patients to improve diagnostic capability in a small volume study [28]. Fluorocholine PET CT outperformed FDG-PET in detecting bone marrow infiltration and bony lesions in patients with myeloma [28]. Whether these results would be used or not for solitary plasmacytomas is not clear but undoubtedly promising. As progress in this area continues, FDG will keep its role in the short and mid-term.

FDG-avidity of the lesions can also serve as a prognostic marker. Alongi et al. have demonstrated that a higher post-radiotherapy maximum standardized uptake value (SUVmax) can portend a poor prognosis. In this trial, 3-year progression-free survival was 28% if the SUVmax level was greater than 4 [7]. Of note, all patients included in this trial were diagnosed with SBP. Albano et al. have investigated prognostic PET CT findings at diagnosis in a mixed group of SBP and EMP patients [29]. The investigators of this trial used quantitative and semiquantitative measures such as SUVlbm (SUV lean body mass), SUVbsa (SUV body surface area), SUVbm (SUV body mass), metabolic tumor volume (MTV), and total lesion glycolysis (TLS). This trial concluded that bone plasmacytomas, SUVlbm >5.2 tumors, and SUVbsa >1.7 tumors progressed to myeloma in a significantly shorter time [29]. FDG-avidity and bone localization simply indicated a higher risk of progression [29].

## 3. Laboratory Studies

Initial laboratory studies are not varied among plasma cell neoplasms. Essential tests that are suggested by the NCCN v2.2025 guidelines include the following [24]:

- CBC, differential, and platelet count;
- Peripheral blood smear;
- Serum BUN/creatinine, electrolytes, liver function tests, albumin, calcium, serum uric acid, serum LDH, and beta-2 microglobulin;
- Creatinine clearance (calculated or measured directly);
- Serum quantitative immunoglobulins, serum protein electrophoresis (SPEP), and serum immunofixation electrophoresis (SIFE);
- Twenty-four-hour urine for total protein, urine protein electrophoresis (UPEP), and urine immunofixation electrophoresis (UIFE);
- Serum free light chain (FLC) assay;
- Unilateral bone marrow aspirate and biopsy, including immunohistochemistry (IHC) and/or multiparameter flow cytometry;
- Plasma cell fluorescence in situ hybridization (FISH) panel on bone marrow [del(13), del (17p13), t(4;14), t(11;14), t(14;16), t(14:20), 1q21 gain/1q21 amplification, 1p deletion];
- NT-proBNP/BNP.

Some patients with SPs have small monoclonal protein in their serum, while SBPs are more secretory than EMPs [8]. A total of 24% to 72% of SBP patients are reported as secretory, while 20% of EMPs have M-protein [8]. Multiparameter flow cytometry is an important tool for detecting clonal plasma cells in the bone marrow, also known as ‘occult disease’ [8]. The presence of these clonal neoplastic cells has prognostic implications; occult disease-positive SBPs more frequently progress to myeloma [8]. Abnormal free light chain ratio can be found in nearly half of the SBP cases and is indicative of larger M-protein [30].

Marrow studies should be performed to exclude multiple myeloma [24]. The following are the IMWG criteria [31]:

- SPs with 10% or more clonal plasma cells in bone marrow are defined as multiple myeloma.
- SPs with less than 10% clonal plasma cells in bone marrow are defined as solitary plasmacytoma with minimal marrow involvement.
- SPs with no clonal plasma cells in bone marrow are defined as solitary plasmacytoma. Plasmacytomas are divided into bone or extramedullary based on the origin site.
- For the multiple myeloma diagnosis, the clonality requirement for marrow plasma cells no longer exists in the NCCN v2.2025 guidelines if a plasmacytoma has been found [24].

### 3.1. Treatment and Response Evaluation

#### 3.1.1. Radiation Therapy

The optimal approach for SPs should include (a) durable long-term local control, (b) minimizing morbidity, and (c) effective pain management. To date, radiation therapy (RT) is the standard of care for SBP and EMP and carries a possibility of cure [9].

The optimal RT dosing is yet to be determined for SP. A recent study conducted in the United States suggested a survival benefit irrespective of tumor diameter when at least 40–45 gray (Gy) radiation was given; this finding calls into question prior reports that advocate lower doses [12]. This large cohort consisted of 9427 patients, with 73% of them having SBP [12]. Radiation alone and with surgery was given to 58% and 20% of SBP patients and 36% and 27% of EMP patients. Combined modality treatment was associated with improved survival compared with radiation alone (181 months vs. 116 months) [12]. Ozsahin et al. demonstrated no benefit of doses greater than 30 Gy, regardless of the tumor size. Tsang et al. reported that doses greater than 35 Gy did not translate into a clinical benefit for <5 cm tumors [13,32]. This diameter-adjusted radiation dosing approach is still endorsed by the recent NCCN guidelines [24]. In 2011, Reed et al. indicated that doses of 40 Gy and higher were associated with 94% local control versus 69% for lower doses [33]. Saba et al. proposed that a radiation dose exceeding 50 Gy confers the greatest benefit for overall survival [10]. Some reports indicate that EMPs are better treated with slightly greater doses of radiation [9,11,34].

The International Lymphoma Radiation Group (ILROG) recommendations are as follows [9]:

- SBPs smaller than 5 cm: 35 Gy.
- SBPs 5 cm and greater: 40–50 Gy.
- EMPs: Doses lower than 40 Gy are not adequate; 40–50 Gy is recommended. Selected low tumor burden patients are set to receive 40 Gy. The ILROG recommends 1.8–2 Gy daily fractions.

The ILROG recommendation for spinal cord compression is as follows [9]:

- 30 Gy in 10–15 daily fractions, 5 fractions/week.

For patients with nerve root compression, systemic steroids in conjunction with radiation is also advocated by the ILROG [9].

Head and neck EMPs can spread to cervical lymph nodes in 15% of cases [35]. The ILROG guidelines recommend against prophylactic irradiation to cervical nodes for Waldeyer’s ring structure-originated EMPs [9]. Except for these tumors, ipsilateral uninvolved cervical lymph nodes may be treated with radiation [9].

Toxicities would be expected, but it is generally well-tolerated. Common acute adverse reactions are mucositis and local erythema. The most common late toxicities are xerostomia and fatigue. Grade 4 toxicity is not expected, while grade 3 toxicity was only 4% in one study [36]. If there is uncertainty about the extent of the bone involvement of SBP, the whole bony compartment, such as the entire spine, can be irradiated without any serious additional toxicity risk [9]. While radiotherapy remains the cornerstone treatment for solitary plasmacytoma, the long-term risk of secondary malignancies should not be overlooked. It was reported that approximately 7% of patients developed secondary malignancies following radiotherapy during long-term follow-up [32]. Although the absolute risk is relatively low, this finding highlights the importance of survivorship surveillance and careful patient selection, especially in younger patients with a longer life expectancy.

#### 3.1.2. Surgery

Surgery alone is not considered a standard therapy according to recent guidelines [20,24]. However, mainly upper and lower airway EMPs can be treated with radical surgery alone with excellent outcomes. According to a retrospective study from the Surveillance, Epidemiology and End Results (SEER) database, surgery alone for EMPs affecting the upper and lower airways is superior in terms of survival to radiation and combined modality. The 5-year relative survival for the surgery-only approach was 96.7% for upper and 100% for lower airway locations in this study. The progression to MM rate was not reported [37]. Conversely, some researchers are advocating to avoid radical surgeries for head and neck EMPs due to the difficulty of obtaining negative surgical margins and the radiosensitivity of these tumors [25]. Goyal et al. reported that genitourinary and gastrointestinal EMPs are most commonly treated with surgery alone, with significantly better outcomes for digestive system tumors [38]. In a recent report, it was reported that radiation-only therapy confers better protection for MM progression than surgery-only therapy [39].

Saba et al. concluded that the median overall survival is 137 months for those treated with surgery alone, which is longer than radiation alone. The surgery alone approach was used more for EMPs than SBPs (23% vs. 8%) [29].

While radiation is considered first for SBPs, certain clinical scenarios would warrant a surgical intervention such as spinal tumors with neurological compromise [25]. Additionally, in one report, surgery was found as a positive prognostic factor for SBPs, mainly located in the spine [40]. Shen et al. demonstrated a median survival of 96 months vs. 77 months (*p* = 0.0005) between surgery and no-surgery spine tumors [40]. The authors of this trial advocate that surgery is associated with a reduction in radiation-resistant clones within the tumor, but the exact mechanism is to be elucidated [40].

#### 3.1.3. Chemotherapy

The role of chemotherapy in SP treatment is a highly controversial issue because most of the studies were performed before the advent of novel agents and with small sample sizes [41]. Ascione et al. recently performed a retrospective analysis of 77 SBP patients, 32 of them treated with chemotherapy. A total of 24 of 32 patients received chemo as adjuvant therapy [41]. The main reason for giving chemo was the inability to reach a CR, and the median time from radiotherapy to chemotherapy was 4.3 months. Immunomodulatory drugs were the most prescribed therapy (87.5% of patients). The 5-year multiple myeloma-free survival (MMFS) rate was 62.9% for patients treated with adjuvant chemotherapy and 41.7% for radiotherapy alone. In this study, chemotherapy-treated patients had more frequently detectable M-protein, thus conferring a higher-risk patient group [41]. Mignot et al. investigated concomitant radiotherapy with lenalidomide and dexamethasone. In this analysis, lenalidomide was administered for 21 days in a 28-day cycle, with a total of 4 cycles, and dexamethasone was routinely added at 40 mg per week. PET CT examination was performed at month 4 of therapy. The MMFS (100% vs. 77.1%) and PFS (81.7% vs. 48.4%) rates were significantly higher than radiation alone therapy [12]. Mheidly et al. found no OS difference between chemo plus radiation and radiation alone, although the PFS benefit of combined therapy was prominent for patients younger than 60 years. These patients’ median PFS was 209 months, and the 5-year PFS was 89%, while the median PFS was 20 months and the 5-year PFS was 46.5% in the radiation alone arm. In this trial, 85% of patients had less than 5% plasma cells on bone marrow specimens, and 85% of patients had detectable serum M-protein. Lenalidomide was not used for any patient, while autologous stem cell transplant and bortezomib-based triplet were the most common modalities, suggesting an important role of bortezomib [42]. An older prospective trial also suggested the benefit of melphalan treatment after radiation for SBPs [43].

IDRIS is a randomized, open-label, multicenter phase 3 study that evaluates the role of adjuvant chemotherapy after radiation for high-risk solitary bone plasmacytomas [44]. High risk was defined as an abnormal free light chain ratio and the existence of clonal plasma cells in the bone marrow [44]. The trial closed early due to the COVID-19 pandemic, but 36 patients were registered [44]. Nine cycles of lenalidomide and dexamethasone regimen were evaluated, and 75% of patients completed lenalidomide, while 67% of patients completed dexamethasone. Lenalidomide was given at a 25 mg/day dose between 1 and 21 days of a 28-day cycle, and dexamethasone was given at 20 mg/week dosing [44]. The chemo arm showed some evidence for a longer PFS (NR vs. 13 months, hazard ratio (HR): 0.32, 95% confidence interval (CI) 0.09–1.07 ) [44].

LENAZART is an open-label, single-center, and single-arm study that evaluates the efficacy of CC-486 (oral formulation of azacytidine), lenalidomide, and radiotherapy [45]. The rationale for this combination is to enhance the antigen presentation on the cell surface since CC-486 upregulates the cancer testis antigen (CTA) and other neo-antigens [45]. Increased antigen presentation leads to the augmentation of antigen-specific immune responses, which are fueled by lenalidomide [45]. The net effect of CC-484, lenalidomide, and RT will be the disruption of the tumor microenvironment and cell death [45]. The results have not been published yet but are eagerly awaited.

### 3.2. Follow-Up, Response Assessment, and Prognosis

According to the NCCN guidelines, response assessment should not occur at less than three months after radiation therapy. Clinicians should be aware that response assessments must not rely solely on imaging techniques; laboratory results should be combined with imaging if the tumor secretes monoclonal protein [24]. The European Expert Panel has defined the response criteria, which remain unchanged today [20]. Since there is no universally accepted concept of ‘higher risk’ beyond the site of origin, consistent follow-up recommendations regarding monoclonal protein are not available in the current literature. The European Expert Panel does not specify the follow-up frequency for monitoring monoclonal protein but acknowledges that serum monoclonal protein levels could remain unchanged in the first months of treatment [20]. The NCCN guidelines recommend measuring serum monoclonal protein every 3–6 months as needed [24]. The MD Anderson Cancer Center solitary plasmacytoma algorithm indicates that the persistence of serum paraprotein after one year necessitates multidisciplinary re-evaluation, including specialists from various disease sites [46]. While the Expert Panel favors FDG-PET CT over MRI for follow-up imaging for all solitary plasmacytomas, the NCCN guidelines primarily recommend FDG-PET CT for extramedullary plasmacytomas [20,24]. The NCCN favors whole-body MRI for the follow-up of solitary bone plasmacytomas over PET CT [24]. Table 3 demonstrates the response definitions for solitary bone plasmacytomas and extramedullary plasmacytomas [20]. The major guidelines are summarized in Table 4.

Prognostic factors that determine overall survival and progression to myeloma can be evaluated at diagnosis and during follow-up. EMPs generally have a better prognosis than SBPs, with median overall survivals reported to be around 89 months for SBPs and 117.3 months for EMPs [47]. Progressing into multiple myeloma is a significant concern when discussing the prognosis of these solitary plasmacytomas (SPs). SBPs progress to multiple myeloma at a rate of 65–84% over 10 years, with a median progression time of approximately 2–5 years [48]. Larger lesions, older age, high M-protein levels at diagnosis, and persistent M-protein after definitive treatment are traditional prognostic variables for solitary bone plasmacytomas [14,49,50]. Genetics play a critical role in myeloma progression, as Yadav et al. proposed [51]. Their retrospective study found that high-risk cytogenetic abnormalities detected by FISH in clonal plasma cells—obtained either directly from the diagnostic tissue or from the bone marrow—are linked to a higher likelihood of myeloma progression [51]. High-risk lesions are defined as del(17p), t(14;16), t(4;14), and/or +1q (gain or amplification) [51]. Patients with high-risk FISH abnormalities experience a significantly shorter median time to multiple myeloma progression (8 vs. 42 months, *p* < 0.001) [49]. Deletion of 17p and +1q abnormalities are among the most common FISH findings associated with faster progression [51]. Although the data suggest a connection between high-risk genetics and myeloma progression, radiotherapy success does not seem to be affected by these genetic features [52]. Fouquet et al. studied the prognostic value of the serum free light chain (sFLC) ratio and FDG-PET CT findings [53]. Multivariate analysis revealed that an initial abnormal involved FLC and the number of hypermetabolic lesions on the initial FDG-PET CT were the most significant prognostic markers; abnormal involved FLC at diagnosis and two or more hypermetabolic lesions on FDG-PET were linked to the shortest median time to myeloma progression of 21 months [53]. Multiparameter flow cytometry also helps assess prognosis in solitary plasmacytoma [54]. Paiva et al. explored how flow cytometry findings relate to progression to myeloma. In this study, all patients had less than 5% plasma cells by microscopy. Clonal plasma cells were detected in 49% of SBPs and 38% of EMPs. Patients who were flow-positive at diagnosis progressed to myeloma more often than flow-negative patients (71% vs. 8%, *p* <0.001). Flow positivity was defined as detecting at least 20 plasma cells by multiparameter flow cytometry with a sensitivity level of 10^−4^ [54].

If left untreated, progression to myeloma will occur in 3 years in 10% of patients. Minimal bone marrow involvement increases this possibility to 60% for SBPs and 20% for EMPs [20]. It has been demonstrated that initial plasmacytoma and subsequent myeloma share the same cytogenetics, which indicates a phylogenetic relationship [55]. These findings raised the idea that SP is an early manifestation of advanced myeloma and triggers early treatment instead of watch and wait [55].

## 4. Conclusions and Future Directions

SBP carries many uncertainties, mainly about optimal management, and is considered a low-risk disease condition; radiation therapy is curative for nearly one-half of patients. Even with radiation therapy, patients presenting with at least one of the high-risk markers have a median time to progression to symptomatic myeloma of roughly 1 year, demonstrating that there is an unmet need for patients who need systemic treatment to prevent further end-organ damage. We believe that the rarity of the disease plays a major role in most of the dilemmas. Larger, well-designed, and prospective trials are urgently needed to clarify the issue further. Immunomodulatory agents such as lenalidomide likely will have a larger role in the treatment of myeloma and likely will be investigated as a part of combination therapy in the future. Other agents that have proven to be effective against myeloma, such as proteosome inhibitors and monoclonal antibodies, are good candidates to be combined with lenalidomide. Other agents such as azacytidine are also showing promise, with results that are underway.

## 5. Limitations

No specific systematic methodology (PRISMA, GRADE, etc.) is used in this review, and this is the major limitation of the paper.

## Figures and Tables

**Table 1:** Diagnostic criteria for solitary plasmacytoma and solitary plasmacytoma with minimal marrow involvement.

Condition	Diagnostic Criteria
Solitary plasmacytoma	Biopsy-proven solitary lesion of bone or soft tissue with evidence of clonal plasma cells. Normal bone marrow with no evidence of clonal plasma cells. Normal skeletal survey and MRI and/or CT of spine and pelvis (except for primary solitary lesion). Absence of end-organ damage such as hypercalcemia, renal insufficiency, anemia, or bone lesions that can be attributed to lymphoplasma cell proliferative disorder.
Solitary plasmacytoma with minimal marrow involvement	Biopsy-proven solitary lesion of bone or soft tissue with evidence of clonal plasma cells. Clonal bone marrow plasma cells < 10%. Normal skeletal survey and MRI and/or CT of spine and pelvis (except for primary solitary lesion). Absence of end-organ damage such as hypercalcemia, renal insufficiency, anemia, or bone lesions that can be attributed to lymphoplasma cell proliferative disorder.

**Table 2:** Differential diagnosis of solitary plasmacytomas.

Diagnosis	Serum Monoclonal Protein	Bone Marrow Cytology	End-Organ Damage	Radiological Work-Up
Solitary plasmacytoma	Not required	Negative	Absent (locoregional manifestations are possible)	No other lesions
Solitary plasmacytoma with minimal bone marrow involvement	Not required	Plasma cell infiltration < 10%	Absent (locoregional manifestations are possible)	No other lesions
Macrofocal myeloma	Not required	Monoclonal plasma cell infiltration < 20%	Possible (mainly bone)	Multiple lesions
Multiple myeloma	Present	Monoclonal plasma cell infiltration > 10%	Present	Other lesions may be present

**Table 3:** Response definitions in solitary plasmacytoma.

Response Class	Definition
Complete response (CR)	Complete disappearance of all previously observed abnormalities on radiographic imaging. For patients with a secretory plasmacytoma, a disappearance of monoclonal protein from serum and/or urine. For SBP, the initial radiological abnormalities on MRI or CT should regress or stabilize during an observation time of at least 12 months to fulfill the requirements for a CR. For EMP, the disappearance of soft tissue mass is required for the definition of CR
Very good partial response (VGPR)	A CR with regard to clinical and radiological signs, but with a positive immunofixation or ≥90% reduction in serum monoclonal protein plus urine monoclonal protein level <100 mg/24 h
Partial response (PR)	A ≥50% decrease in serum and/or urine monoclonal protein. For non-secretory SP, radiological features (MRI/CT) or local assessment is needed. In EMP patients, a 30% decrease in the diameter of target lesions should be observed
Stable disease (SD)	Insufficient shrinkage to qualify for PR nor sufficient increase to qualify for PD
Progressive disease (PD)	The development of new lesions or an increase of at least 20% in the size of existing lesions, the appearance of a myeloma defining event, and finally an increase of >25% from the lowest response value in serum and/or urine monoclonal protein

**Table 4:** Selected management overviews from professional organizations and institutions.

Publication	Proposed Management Strategy
NCCN Guidelines [24]	40–50 Gy in 1.8–2.0 Gy fractions radiotherapy with possible dose reductions to 35–40 Gy for tumors < 5 cm, add surgery if the tumor structurally unstable or causes neurologic compromise due to mass effect. Response should be assessed at a minimum of 3 months after radiotherapy. Then, yearly imaging with the same technique that was used for diagnosis for at least 5 years. Head and neck plasmacytomas may be followed less frequently after 3-month assessment. Whole-body MRI is favored for SBPs; PET CT is favored for EMPs. Biochemical follow-up not mandated, only as needed.
MD Anderson Cancer Center Algorithm [46]	35–45 Gy radiotherapy, regardless of site, and 35 Gy dose is favored for tumor diameter < 5 cm. Response evaluation is to be done after 3 months of radiation and should include imaging and biochemical tests (beta-2 microglobulins, LDH, serum and urine immunofixation and protein electrophoresis, quantitative immunoglobulins). PET CT is favored, regardless of site, every 3 months until complete metabolic response. Complete response does not warrant disappearance of tumor, although disappearance of paraprotein and normalization of serum free light chain is mandated. Persistent positive imaging after 6 months of treatment and/or persistent paraprotein after 1 year of treatment requires a multidisciplinary evaluation with the disease site specialists.
European Expert Panel Recommendations [20]	A total 40–50 Gy radiation dose is advocated. Surgery is reserved for neurological compromise due to mass effect and impending/existing pathological fractures. Adjuvant chemotherapy and antiresorptive therapy is not routine but may be considered on a case-by-case basis (for >5 cm tumors and patients with osteoporosis on dual energy X-ray absorptiometry). Response assessment should be performed by serum/urine parameters, and for soft tissue plasmacytomas, RECIST criteria are added to response definitions (Table 3). Time frames are not given for the follow-up schedule; preferentially the same imaging technique should be employed throughout the follow-up period.

## Data Availability

No new data were created or analyzed in this study.
